# MiR-146a Ameliorates Hemoglobin-Induced Microglial Inflammatory Response via TLR4/IRAK1/TRAF6 Associated Pathways

**DOI:** 10.3389/fnins.2020.00311

**Published:** 2020-04-03

**Authors:** Guang-Jie Liu, Qing-Rong Zhang, Xuan Gao, Han Wang, Tao Tao, Yong-Yue Gao, Yan Zhou, Xiang-Xin Chen, Wei Li, Chun-Hua Hang

**Affiliations:** ^1^Department of Neurosurgery, Nanjing Drum Tower Hospital, The Affiliated Hospital of Nanjing University Medical School, Nanjing, China; ^2^Department of Neurosurgery, Jinling Hospital, School of Medicine, Southern Medical University (Guangzhou), Nanjing, China; ^3^Department of Neurosurgery, Nanjing Drum Tower Hospital, Clinical College of Nanjing Medical University, Nanjing, China

**Keywords:** subarachnoid hemorrhage, microglial polarization, miR-146a, neuro-inflammation, TRAF6, IRAK1

## Abstract

Microglial activation and sustained inflammation in the brain can lead to neuronal damage. Hence, limiting microglial activation and brain inflammation is a good therapeutic strategy for inflammatory-associated central nervous disease. MiR-146a is a promising therapeutic microRNA, since it can negatively regulate the inflammatory response. We thus investigated the expression changes of miR-146a after experimental induction of a subarachnoid hemorrhage (SAH) *in vivo* and *in vitro*, and we assessed the anti-inflammatory effects of miR-146a in microglial cells *in vitro*. Primary microglial cells were preincubated with miR-146a before hemoglobin (Hb) treatment. The results indicated that miR-146a decreased gene expression of Hb-induced pro-inflammatory cytokines (TNF-α and IL-1β) and phenotype-related genes (iNOS and CD86) through IRAK1/TRAF6/NF-κB or MAPK signaling pathways, suggesting its pro-resolution activity in microglia. However, contrary to the LPS-induced microglia or macrophage activation model, we did not observe an elevation in miR-146a after activation. Overall, our findings demonstrated that miR-146a was involved in the regulation of brain inflammation and could be considered a novel therapeutic agent for treating brain inflammation.

## Introduction

Aneurysmal SAH is a devastating disease. EBI is thought to be the main cause of poor outcomes after SAH (Fujii et al., 2013). Inflammation, as a critical role in EBI, has a great impact on the pathology and outcome of SAH, such as blood brain barrier interruption, vasospasm, oxidative stress, or even neuronal death (Miller et al., 2014). Therefore, many efforts have been made to regulate inflammation as a method to avoid excessive damage after SAH.

Microglia are the main glial cells responsible for regulating inflammation. The immune response can be characterized by different cellular phenotypes that are triggered by various factors, and phenotype switching can occur with the progression of disease (Aguzzi et al., 2013; Hu et al., 2015; Lan et al., 2017). Among those phenotypes, pro-inflammatory polarization is characterized by the release of many pro-inflammatory factors (TNF-α, IL-1β, CD86, and iNOS), while anti-inflammatory polarization present with the expression of many anti-inflammatory or phagocytosis-related proteins (IL-4, IL-10, Arg1, and CD206). Our previous studies have demonstrated that inhibiting pro-inflammatory polarization and promoting anti-inflammatory polarization improved neurological function after SAH (Sun et al., 2013; Zhang et al., 2016; Tao et al., 2019). In pro-inflammatory polarization, the TLR4/NF-κB signaling pathway is significantly activated (Hanafy, 2013; Lu et al., 2018), making it the dominant target of drugs for attenuating inflammation (Sheedy et al., 2010; Rahimifard et al., 2017).

MicroRNAs are small (∼22 nt), non-coding RNA molecules. They function at the posttranscriptional stage and bind to the 3’ untranslated regions (3’ UTRs) of target messenger RNA transcripts (mRNAs), usually leading to gene silencing (Bi et al., 2009; Xie et al., 2013). MiR-146a was found to play an important role in regulating of the inflammatory process. Many cell types under different pathologic conditions are regulated by miR-146a, such as human retinal endothelial cells (Cowan et al., 2014), macrophages (Alam and O’Neill, 2011), and human gingival fibroblasts (Xie et al., 2013). For disease such as Alzheimer’s disease, miR-146a was also found to be a potential treatment target (Wang et al., 2012). Among the functional mechanisms of miR-146a, TRAF6 and IRAK1 are two key recognized target genes in the TLR4 signaling pathway (Taganov et al., 2006), i.e., miR-146a functions as a dominant negative regulator of TLR4/IRAK1/TRAF6/NF-κB signal transduction (Ma et al., 2011; Quinn and O’Neill, 2011; Saba et al., 2014).

Thus, we conducted a study to observe the expression pattern of miR-146a and then explored whether the exogenous addition of miR-146a could have an impact on microglial polarization resulting in a neuronal protection.

## Results

### MiR-146a Was Reduced in an Experimental Subarachnoid Hemorrhage Model *in vivo* and in Hb-Stimulated Primary Microglia *in vitro*

**FIGURE 1:**
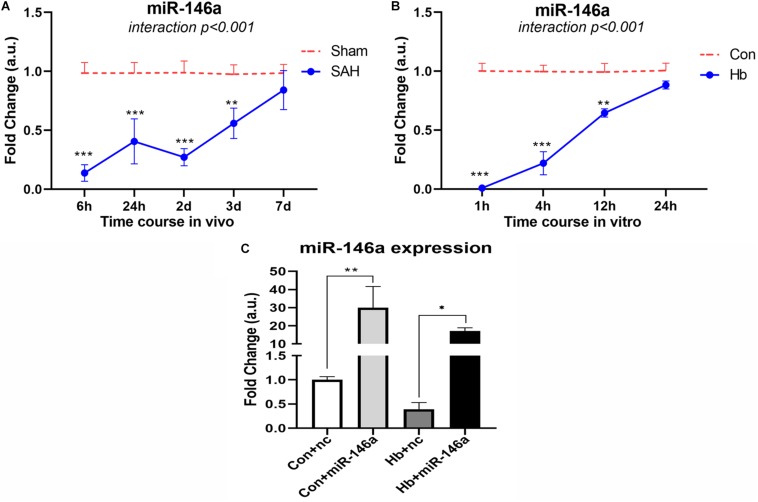
MiR-146a expression after experimental SAH *in vivo* and *in vitro*. **(A)** Time course of miR-146a expression after experimental SAH *in vivo* from 6 h to 7 days (*n* = 3). **(B)** Time course of miR-146a expression after Hb-stimulated microglia *in vitro* from 1 to 24 h (*n* = 3). **(C)** Microglial miR-146a expression change after exogenous addition of its mimics (*n* = 3). Con+nc: control+miR-146a mimic negative control; Con+miR-146a: control+miR-146a mimic; Hb+nc: Hb+miR-146a mimic negative control; Hb+miR-146a: Hb+miR-146a mimic. Data were analyzed by two-way ANOVA (interaction: time*treatment) for **A** and **B**; one-way ANOVA followed by *post hoc* Tukey’s test for **C**. *p < 0.05, **p < 0.01, and ***p < 0.001. *n* = number of rats or independent cell samples.

To determine the endogenous expression pattern of miR-146a after SAH, we assessed levels over a time course in an animal model *in vivo* and in Hb-activated microglia *in vitro*. *In vivo*, miR-146a was evidently reduced at an early stage of 6 h, and the reduction was sustained for 2 days from the onset of experimental SAH (Figure 1A). It then increased gradually from 3 to 7 days, while the levels were still lower than those of the sham group. A similar expression pattern was observed in primary microglia *in vitro* (Figure 1B). We detected from 1 to 24 h, that miR-146a was severely attenuated, and then it was subsequently elevated but did not reach the level of the control at 24 h.

### MiR-146a Levels Were Greatly Increased After Exogenous Addition

We transfected miR-146a mimics into microglia *in vitro*, and after 12 h we stimulated them with Hb for 4 h to determine the transfection effect (Figure 1C). The results showed that with the addition of mimics, the level of miR-146a in microglia was dramatically increased. There was approximately 30-times more miR-146a in the Con+miR-146a (control+miR-146a mimic) group than there was in the Con+nc group (control+miR-146a mimic negative control), and the Hb+miR-146a (Hb+miR-146a mimic) group exhibited approximately 30 times more than that of the Hb+nc (Hb+miR-146a mimic negative control) group. This indicated that exogenous addition of miR-146a was successful.

### Hemoglobin Induced Strong Pro-Inflammatory Polarization of Primary Microglia

**FIGURE 2:**
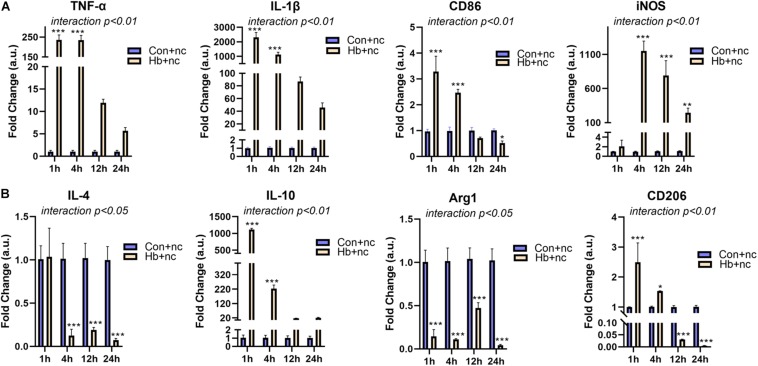
Proinflammatory response and phenotypic modification of Hb-induced primary microglia. Primary microglial cells were treated by 20 μM Hb and a miR-146a negative control (nc, 50 nM) from 1 to 24 h. **(A)** mRNA expression of the proinflammatory markers TNF-α, IL-1β, CD86, and iNOS (*n* = 3). **(B)** mRNA expression of the antiinflammatory markers IL-4, IL-10, Arg1, and CD206 (*n* = 3). Data are presented as the fold change calculated relative to the control+nc group (baseline = 1). Values are means ± SD (*n* = 3). Data were analyzed by two-way ANOVA (interaction: time*treatment) followed by the Sidak test for multicomparison. *p < 0.05, **p < 0.01, and ***p < 0.001. *n* = independent cell samples. nc miR-146a mimic negative control.

We next examined mRNA expression of microglial polarization markers (pro-inflammatory markers: TNF-α, IL-1β, CD86, and iNOS; anti-inflammatroy markers: IL-4, IL-10, Arg1, and CD206) *in vitro* under Hb stimulation. For proinflammatory markers, TNF-α, IL-1β (cytokines), and CD86 (cell surface marker) showed similar patterns, reaching peaks at 1 h and then gradually decreasing. iNOS (intracellular marker) levels peaked at 4 h and subsequently decreased (Figure 2A). For anti-inflammatory markers, IL-10 (cytokines) and CD206 (cell surface marker) were elevated and peaked at 1 h, while IL-4 (cytokines) and Arg1 (intracellular marker) were sustained at significantly reduced levels for 24 h (Figure 2B).

### MiR-146 Attenuated Hemoglobin-Induced Inflammation and Slightly Promoted Anti-Inflammatory Phenotype Switching in the Late Phase of the Time Course

**FIGURE 3:**
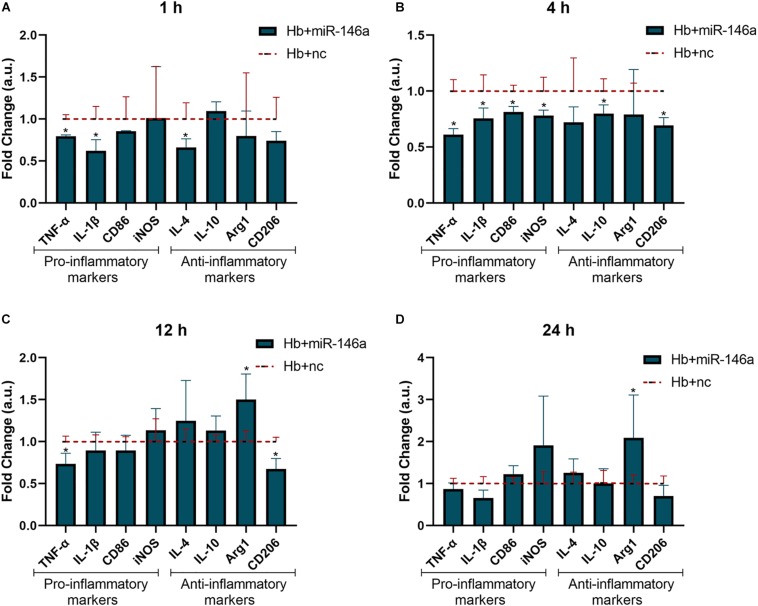
Effect of miR-146a on the Hb-induced inflammatory response and microglial polarization at different time points. Microglia were pretreated with miR-146a (50 nM) for 12 h and then Hb (20 μM) was added for 1 to 24 h. Data are presented as the fold change calculated relative to the Hb+nc group (baseline = 1). Values are means ± SD (*n* = 3). Statistical analysis was performed using a Mann–Whitney *U*-test for each gene at each time point. *p < 0.05. nc miR-146a mimic negative control.

To investigate the potential anti-inflammatory role of miR-146a on microglia in response to Hb stimulation, we observed the mRNA expression of the above pro/anti-inflammatory factors at 1, 4, 12, and 24 h (Figure 3). The alterations of TNF-α and IL-1β were the most significant results; they were obviously attenuated at 1 h (Figure 3A), and the reduced levels were maintained until 24 h (Figure 3D) compared to that of Hb+nc group even if their levels were higher than con+nc group (combined with Figure 2). The phenotype markers CD86 and iNOS were reduced at 4 h (Figure 3B), but tended to increase later. Some anti-inflammatory markers such as IL-4 and CD206 were also inhibited by the addition of miR-146a in 1 or 4 h, and then IL-4 gradually increased while CD206 remained reduced. IL-10 was attenuated at 4 h and did not differ evidently from that of the Hb+nc group later. Arg1 was elevated at 12 h (Figure 3C) and sustained to 24 h (Figure 3D). These results demonstrated that cellular responses to inflammatory stimuli were very complex.

**FIGURE 4:**
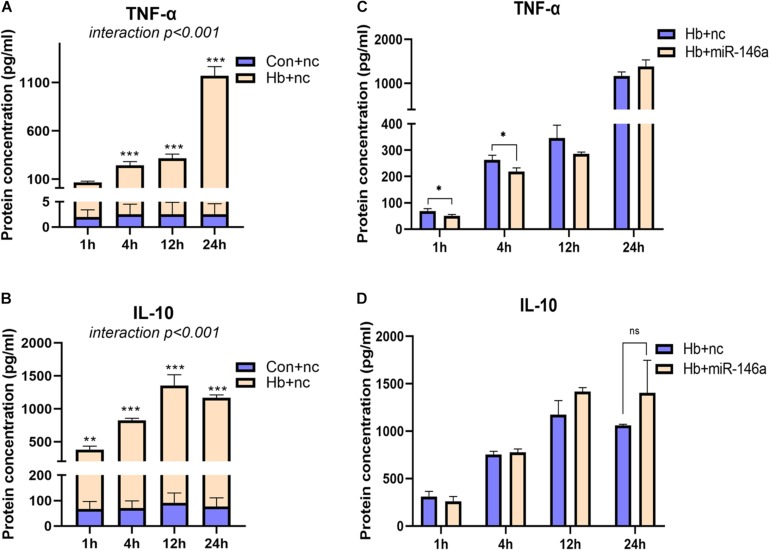
Time-dependent effect of miR-146a on Hb-induced TNF-a and IL-10 release. Cell culture supernatant was collected for examination. **(A,B)** Primary microglial cells were treated with 20 μM Hb from 1 to 24 h. **(C,D)** Microglia were pretreated with miR-146a (50 nM) or a miR-146a negative control (nc, 50 nM) for 12 h, and then Hb (20 μM) was added for 1–24 h. Data are presented as the real protein concentration **(A,B)** or fold change calculated relative to the Hb+nc group (**C,D** baseline = 1). Values are means ± SD (*n* = 3). Statistical analysis was performed using a two-way ANOVA (interaction: time^∗^treatment) which was followed by the Sidak tests for multicomparison **(A,B)** or one-way ANOVA followed by *post hoc* Tukey’s tests for each gene. ^∗^*p* < 0.05, **p < 0.01, ***p < 0.001. nc miR-146a mimic negative control.

For TNF-α and IL-10, we also examined their protein levels; TNF-α was markedly induced by Hb and increased from 1 to 24 h (Figure 4A). MiR-146a reduced its protein levels at 1 and 4 h, not at 12 and 24 h, and even a small elevation was observed at 24 h (Figure 4C). IL-10 was also increased by Hb but had a reduction at 24 h, while miR-146a had no significant effect on it (Figures 4B,D).

### TLR4/IRAK1/TRAF6 mRNA Expression Was Regulated by miR-146a

**FIGURE 5:**
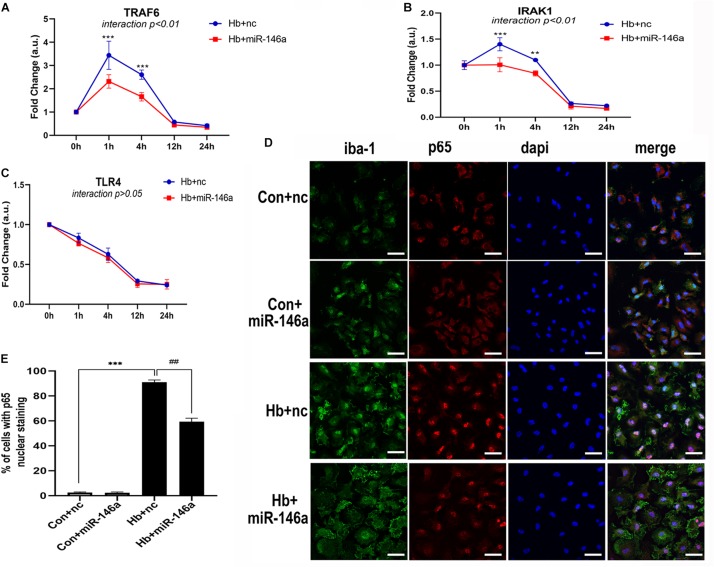
MiR-146a effects on the TLR4/TRAF6/IRAK1/NF-κB signaling pathway. **(A–C)** Primary microglia were pretreated with miR-146a (50 nM) or miR-146a negative control (nc, 50 nM) for 12 h and then Hb (20 μM) was added for 1–24 h. mRNA expression of TLR4, TRAF6, and IRAK1 was presented as the fold change calculated relative to the con+nc group (*n* = 3). **(D,E)** Representative images of immunofluorescence of p65 nuclear translocation and corresponding quantification (*n* = 3). Values are means ± SD. Statistical analysis was performed using a two-way ANOVA (interaction: time^∗^treatment) **(A–C)** or one-way ANOVA followed by *post hoc* Tukey’s tests for multi-comparison. **p < 0.01, ***p < 0.001, and ##*p* < 0.01. nc miR-146a mimic negative control. Bar = 40 μm.

We next investigated the influence of miR-146a on the mRNA levels of members of the TLR4/IRAK1/TRAF6 signaling pathway. According to the results, TRAF6 and IRAK1 were markedly reduced by miR-146a and the main time at which a difference in regulation phase was observed was before 12 h (Figures 5A,B). TLR4 showed a reduction tendency following miR-146a treatment; however, there was no significant difference (Figure 5C). Then, we detected the effect of miR-146a on P65 translocation to the nucleus (indication of NF-κB activation) with IF (Figure 5D). Hb induced a rapid and large amount of p65 nuclear translocation, which was decreased by addition of miR-146a (Figures 5D,E).

### NF-κB/MAPK Signaling Pathway Activation Was Reduced by miR-146a

**FIGURE 6:**
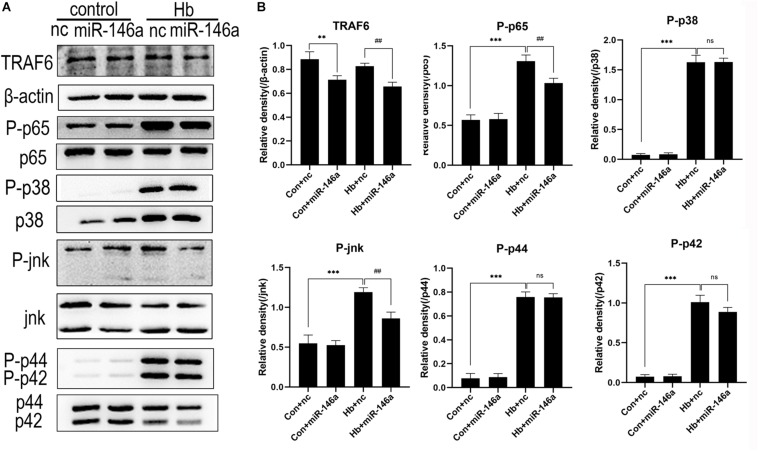
Effects of miR-146a on the expression of proteins in the TLR4/TRAF6/IRAK1/NF-κB signaling pathway. **(A)** Western blotting results of these proteins. **(B)** Corresponding quantification analysis of **A**. Primary microglial cells were preincubated with miR-146a (50 nM) or miR-146a negative control (nc, 50 nM) for 12 h, and then Hb (20 μM) was added for 15 min. TRAF6 levels were normalized to β-actin expression, and P-p65, P-p38, P-jnk, P-p44, and P-p42 were normalized to their corresponding non-phosphorylation proteins. Values are means ± SD (*n* = 3). Statistical analysis was performed using one-way ANOVA followed by *post hoc* Tukey’s test for multicomparison. **p < 0.01, ***p < 0.001, ##*p* < 0.01, and ns = no significance. nc miR-146a mimic negative control.

NF-κB is a key transcriptional factor triggered by the TLR4 signaling pathway, and it promotes the expression of many proinflammatory cytokines and chemokines in many immune cell types including microglia (Doyle and O’Neill, 2006). To further confirm our hypothesis that miR-146a inhibits TLR4 signaling pathway activation, we detected the downstream proteins involved in TRAF6 and NF-κB activation by Western blotting. Compared to the Con+nc group, TRAF6 protein expression was reduced after the addition of miR-146a, while there was no significant change in the phosphorylation of p65 protein (activation of p65). Hb greatly induced phosphorylation of p65 over what was observed in the Con+nc group. MiR-146a decreased the phosphorylation of p65 (Figures 6A,B).

In addition to NF-κB, the MAPK pathway is known to be involved in TLR4-mediated microglial inflammatory responses. We therefore detected the proteins jnk, p44/42 and p38 and their phosphorylation conditions. The three protein levels were markedly elevated after Hb stimulation, as revealed by comparing the Con+nc group with the Hb+nc group. MiR-146a reduced the phosphorylation of jnk; however, it had almost no impact on the phosphorylation p44/42 or p38 (Figure 6).

### Proinflammatory Microglial Activation-Induced Neuronal Death Was Inhibited by miR-146a

**FIGURE 7:**
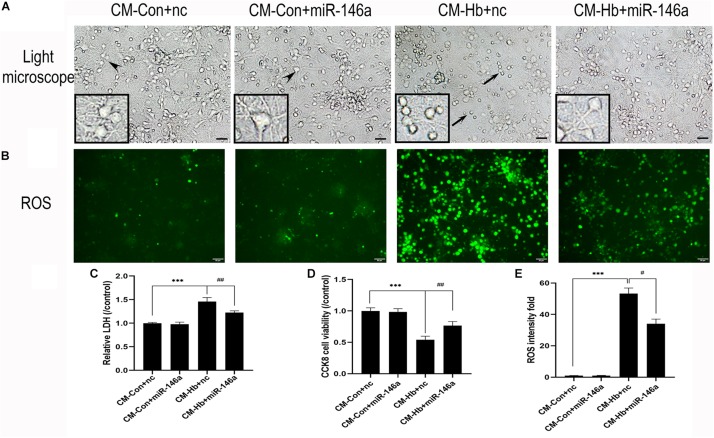
Effect of conditioned medium (CM) from microglia on primary neurons. Primary neurons were treated with CM for 12 h. **(A)** Representative microscopy image of neurons. **(B)** ROS production observed by green fluorescence. **(C–E)** LDH release (*n* = 6), CCK8 cell viability (*n* = 6), and ROS production (*n* = 6) relative to the CM-Con+nc group. Values are means ± SD. Statistical analysis was performed using one-way ANOVA followed by post hoc Tukey’s tests for multicomparison. ***p < 0.001, #*p* < 0.05, and ##*p* < 0.01. Black arrow: neuron soma shrinkage and neurite degeneration and arrowhead: normal neurons. miR-146a mimic negative control. Bar = 15 μm.

Neuronal death is a severe pathological process in many acute or chronic diseases of central nervous system. For SAH, inflammation is a critical cause of neuronal death. Thus, we used conditioned medium (CM) from activated microglia to culture primary neurons, enabling us to observe whether miR-146a could reduce neuronal damage. CM from the Hb+nc group resulted in significant damage to neurons, characterized by soma shrinkage or neurite degeneration (black arrows) (Figure 7A). ROS production (Figures 7B,E), and LDH release (Figure 7C) were also elevated in the CM-Hb+nc group compared to the levels in the CM-Con+nc group, while cell viability was reduced (Figure 7D). However, in the CM-Hb+miR-146a group, neuronal damage was decreased, ROS production was reduced, LDH release was reduced, and cell viability was elevated.

## Discussion

In this study, we demonstrated for the first time that miR-146a is reduced after SAH *in vivo* and *in vitro*. Exogenous addition of miR-146a serves as a potent inhibitor of Hb-induced TNF-α and IL-1β gene expression and a regulator of the microglial phenotype *in vitro*. Our results deepen our comprehension on the beneficial effects of miR-146a in the central nervous system.

The *in vitro* experiment could not fully mimic SAH *in vivo*. MiR-146a fold changes were still low until 2 or 3 days, compared to that of primary microglial result recovered at 24 h. Several reasons might account for this. The *in vivo* circumstance, composed by various cell types accompanied with numerous inter-cellular interactions, is far too complex for the *in vitro* experiments to replicate equivalent results. Studies concerning miR-146a in the central nervous system are scarce, while several articles have shined some light on it. Some studies indicated that miR-146a is not just exclusively expressed in microglia, also in astrocytes (Aronica et al., 2010; Iyer et al., 2012; Sison et al., 2017). In addition to that, miR-146a also has impact on neurons (Wang et al., 2014; Jia et al., 2016; Li et al., 2017), oligodendrocytes (Zhang et al., 2017), and endothelial cells (Vasa-Nicotera et al., 2011). Instead of exploring the whole intricate functions of miR-146a, we are particularly interested in how would the miR-146a influence microglial functions.

Subarachnoid hemorrhage is characterized by sterile inflammation, which is different in some ways from LPS-induced inflammation. In our current study, Hb induced a rapid elevation of pro-inflammatory cytokines, which was similar to the results of LPS stimulation and was consistent with previous *in vivo* and *in vitro* studies (Lin et al., 2012; Gram et al., 2013; Kwon et al., 2015). Nevertheless, miR-146a expression was different. Several studies indicated that miR-146a was elevated after LPS stimulation. LPS resulted in rapid elevation of the expression of miR-146a in human monocytic THP-1 cells and BV2 cells (Taganov et al., 2006; Rey et al., 2016). Our study demonstrated that miR-146a was downregulated by Hb stimulation, indicating a different response due to the differences in cell types or stimulation. In LPS-stimulated THP-1 cells and BV2 cell models, further investigation of miR-146a suggested that the induction is NF-κB dependent. Furthermore, TRAF6 and IRAK1, two critical molecules of the TLR4/NF-kB pathway, are confirmed to be direct targets of miR-146a. This suggests a negative regulatory loop in which NF-kB activation upregulates the miR-146 gene, while the latter downregulates IRAK1 and TRAF6 to reduce the activity of NF-kB (Taganov et al., 2006; Saba et al., 2014). However, although Hb did not induce elevation of miR-146a in primary microglia, its regulation of the inflammatory response was evident in our results.

Microglial polarization has been shown to be related to cell functions such as motility, phagocytosis, synapse reconstruction, or antigen presentation (Hanisch, 2013). We showed that miR-146a possibly contributed to microglial phenotype modification. In the scenario of pro-inflammatory polarization, TNF-α, IL-1β, CD86, and iNOS were significantly attenuated at 1 or 4 h until finally recovered to baseline at 12 or 24 h. For anti-inflammatory polarization markers, IL-4 and IL-10 showed no significant elevation after the addition of miR-146a; contrarily, they were attenuated in the early phase (1 or 4 h). Arg1 was not elevated until in the late phase. Most prominently, CD206 was fundamentally inhibited by miR-146a throughout the experimental time course. This receptor is mainly engaged in phagocytosis (East, 2002), and the reason for its continuous attenuation by miR-146a needs further exploration. IL-10 and CD206, elevated after Hb stimulation, could be inhibited by miR-146a possibly because they were partly regulated by TRAF6. Nevertheless, greater number of samples and more additional polarization markers should be introduced to ensure a more promising explanation. Thus, we conclude that the dominant effect of miR-146a on microglia is pro-inflammatory polarization inhibition, as for the influence on microglial anti-inflammatory polarization, further experiments need to be conducted.

We finally detected the expression of proteins involved in the NF-κB/MAPK signaling pathways and of pro-inflammatory cytokines (TNF-α/IL-10). The TRAF6-NF-κB and JNK were obviously inhibited, but P38 or P44/42 were not. One possible explanation is that some other signaling pathways are engaged in the activation of P38 or P44/42 proteins. TNF-α was significantly attenuated in the early stage (1 and 4 h) but not at 24 h. This phenomenon was somewhat inconsistent with the change in mRNA expression. Some posttranslational modifications might be involved in this phenomenon. Hence, time of miR-146a treatment should be considered to ensure its good effect in future investigations.

The effect of CM from activated microglia on primary neurons indicated a good protection effect on microglia from intermediate damage. The neurons cultured in CM might suffer from two damage factors: Hb exposure and inflammatory stimuli. Therefore, it was not clarified whether miR-146a could shield neurons from Hb-induced damage. As our results verified, the inhibition of the inflammatory response could be if not all, part of the reason, while other remaining potential factors also need to be assessed in the future. The results of our *in vitro* experiments indicated that miR-146a could be used not only for treating SAH also for some other hemorrhage or ischemia-associated diseases in central nervous system, such as cerebral hemorrhage, traumatic brain injury, or ischemic stroke, because TLR pathways are widely activated in the pathological process (Wang et al., 2013; Shi et al., 2019); however, miR-146a related studies in these areas are limited.

**FIGURE 8:**
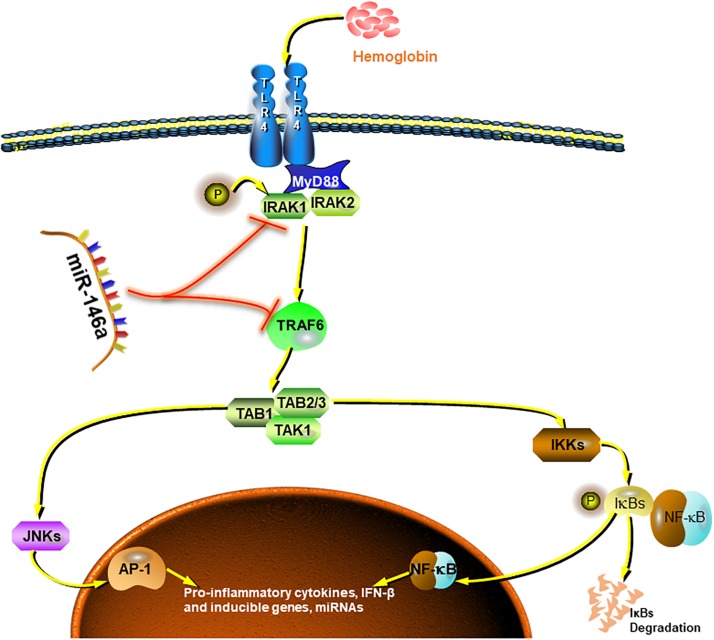
Summarized picture of the effects of miR-146a in this study. MiR-146a attenuates the Hb-induced microglial inflammatory response through TRAF6/IRAK1 inhibition.

Collectively, our preliminary study demonstrated good inhibition by miR-146a of pro-inflammatory polarization of microglia and, to some extent, the promotion of phenotype modification (Figure 8). These effects could attenuate microglia-induced neuron loss and thus exerting neuroprotective effects. However, due to the finite efficacy of *in vitro* experiment and limited sample size, further attentively designed cellular and animal experiments are needed to get more thorough understanding of the underlying mechanisms.

## Materials and Methods

### Experimental Protocol

Experiment 1 was performed *in vivo* as follows: 30 rats were divided into 10 groups: sham group (*n* = 3 for each time point), SAH 6-h group (*n* = 3), SAH 24-h group (*n* = 3), SAH 2-day group (*n* = 3), SAH 3-day group (*n* = 3), and SAH 7-day group (*n* = 3). Total RNA was collected at different time courses, and miR-146a was analyzed.

Experiment 2 was performed *in vitro* as follows. Primary microglia were cultured for three independent experiments: (a) time course analysis of Hb-induced pro-/anti-inflammatory marker expression between 1 and 24 h; (b) analysis of the effects of miR-146a on the expression of the above markers after different time course treatments with Hb; and (c) identification of the signaling pathway regulated by miR-146a.

Experiment 3 was performed *in vitro* to determine the effect of CM from microglia on primary neurons. After sufficient treatment, neuron viability were assessed by CCK8 assays (*n* = 6), LDH assays (*n* = 6), and analysis of ROS production (*n* = 3).

### Animal Experiments

32 male Sprague–Dawley rats (RRID: RGD_10395233) weighing 280–320 g were purchased from Animal Core Facility of Nanjing Medical University. Our experiment was approved by the Experimental Animal Ethics Committee of Nanjing Drum Tower Hospital, approved number: 2018020003. Rats were kept in a comfortable environment equipped with a constant temperature of 26 ± 2°C and 12-h light–dark cycles. During the experiment, rats were housed four per cage (57^∗^36^∗^14.5 cm) and provided with free access to water and a standard chow diet. Each animal’s health condition was estimated every 24 h.

The SAH endovascular perforation model in rats was performed according to previous article (Sehba, 2014). Briefly, rats were transorally intubated and ventilated mechanically during the operation period with 3% isoflurane anesthesia. A 4-0 monofilament nylon suture was sharpened and inserted into the right internal carotid artery rostrally from the external carotid artery and punctured the bifurcation of the anterior and middle cerebral arteries. Rats in the sham-operated group underwent the same procedures but the suture was withdrawn without perforated. Aluminum polyester foil blanket was used during and after surgery until rats recovered from anesthesia. Rats were free to water, jelly, or batter of chow diet after surgery.

At each time point, rats were killed by isoflurane anesthesia followed by decapitation and then the brains were removed. The basal cortex tissue was collected and stored at −80°C for further examination.

### Primary Microglial Cell Culture and Stimulation

Primary microglial cells were cultured as described in a previous article (Lian et al., 2016). Briefly, the cerebral cortex was obtained from the brains of neonatal (1 day) mice. After carefully removing the meninges, brain tissue was digested with 0.25% trypsin (Gibco, United States) for 10 min at 37°C. Subsequently, the pellet was triturated with 6 ml of warm culture media with a 1 ml pipet tip. The suspension was filtered through a 70-μm strainer (Millipore, Sigma) and centrifuged at 1000 r/min for 5 min. The remaining cells were resuspended in Dulbecco’s modified Eagle’s medium (DMEM, Gibco, United States) with the addition of 10% fetal bovine serum (FBS, biological industries, United States), and then cells were seeded in flasks. Three days after the glial cultures reached confluency, the flasks were shaken for 2 h at 340 r/min. The floating cells were collected and seeded in plates to obtain microglia.

After seeding on plates for approximately 1–2 days, microglia presented a resting state; then, miR-146a was added to the medium at a concentration of 50 nM. After 12 h, Hb (20 μM) was added and incubated for 1, 4, 12, and 24 h. At each time point, cells were collected for further experiments.

### Primary Neuron Culture

For neuron culture, the cortex was obtained from mice at embryonic day 15. The next steps were similar to primary microglia culture. After removing the meninges, digestion, titration, and centrifugation were performed, and neurons were seeded on poly-D-lysine precoated plates at a density of 6.0 × 10^5/ml and were incubated at 37°C and 5% CO2. Four hours later, the medium was completely replaced with neurobasal medium containing 2% B27 and 1% GlutMax (Gibco, United States). Neurons were available for use after 7 days of *in vitro* culture (Ferreira et al., 2014).

### MiR-146a Isolation and Quantitative PCR

Quantitative PCR was performed with a m*iDETECT A* Track^TM^ system (Ribobio, Guangzhou, China). MiRNA qRT-PCR Primer Sets (one RT primer and a pair of qPCR primers for each set) specific for miR-146a were obtained from this system. All steps were conducted according to the manufacturer’s instructions. Total RNA was isolated using TRIzol reagent (Invitrogen, United States). MicroRNAs were reverse transcripted after treatment with a method to achieve poly (A) addition. The qPCR recycling program was 95°C for 2 s and 60°C for 30 s, which was performed with SYBER Green mix (Roche, Switzerland) using the a PCR instrument (Applied Biosystems, United States). Each reaction was performed in duplicate and contained a negative control reaction. Data were analyzed by the 2^–ΔΔ*Ct*^ method and U6 was used as an endogenous control.

### Western Blotting Analyses

Cells cultured in six-well plates were washed twice with phosphate-buffered saline (PBS) and lyzed in 100 μl of lysis buffer (Thermo Fisher Scientific, MA, United States) per well. Equal amounts of protein extracted from cells were run on a 10% SDS polyacrylamide gels and then were transferred to a polyvinylidene difluoride membrane (Millipore, Darmstadt, DE, United States). Membranes were blocked for 1 h with 5% skim milk and then were incubated at 4°C overnight with specific antibodies against phosphorylated or total forms of the following antibodies (all purchased from Cell Signaling Technology, United States): jnk (1:2000, cat. No. 4668S), P-jnk (cat. No. 81E11), p65 (1:1000, cat. No. D14E12), P-p65 (1:1000, cat. No. 93H1), p44/42 (1:1000, cat. No. 137F5), P-p44/42 (1:1000, cat. No. D13.14.4E), p38 (1:1000, cat. No. D13E1), P-38 (1:1000, cat. No. D3F9), TRAF6 (1:2000, 66498-1-Ig, Proteintech, Wuhan, China), and β-actin (1:2000 cat No. BS6007M, Bioworld Technology, Minneapolis, MN, United States). Membranes were washed three times with Tris-buffered saline containing Tween (TBST) and were subsequently incubated at room temperature for 1 h with the corresponding horseradish peroxidase (HRP)-conjugated IgG (cat No. BS13278 or BS30503, Bioworld Technology, Minneapolis, MN, United States). Protein signals were developed with an enhanced chemiluminescence solution (cat No. 46640, Thermo Fisher Scientific, Waltham, MA, United States). Band gray intensity was quantified with ImageJ software (RRID: SCR_003070).

### ELISA

Primary microglial cells (6 × 10^5 cells/well) that were in 12-well plates preincubated with miR-146a for 12 h, which was followed by treatment with Hb (20 μM) for 1, 4, 12, and 24 h. The culture medium was collected and centrifuged, and the supernatants were stored at −80°C until analysis. The levels of IL-10 and TNF-α were determined by ELISA according to the manufacturer’s instructions. A standard curve was generated using the mouse IL-10 and TNF-α standards (concentration range 0–1000 pg/ml) that were provided in the kits for each assay.

### Quantitative PCR (qPCR)

Primary microglial cells (6 × 10^5 cells/well) were seeded in 12-well plates. The cells were preincubated with miR-146a for 12 h, which was followed by Hb (20 μM) stimulation for 1, 4, 12, and 24 h. Total RNA was extracted using TRIzol reagent according to the manufacturer’s instructions. The purity and concentration of total RNA were quantified with a BioPhotometer (Eppendorf, Germany). Subsequently, total RNA (1.0 μg) was reverse-transcribed to generate cDNA using a reverse transcription mix (Vazyme, Nanjing). qPCR was performed using a PCR system (Applied Biosystems, United States) with a SYBER Green mix (Roche, Switzerland). The primers for all genes are shown in Table 1. GAPDH was used as an internal control to normalize the expression of each gene. The relative expression of each gene in every group was calculated using the 2^–ΔΔ*Ct*^ method.

**TABLE 1:** Primer pairs used in quantitative PCR.

Primer name		Sequence
IL-1β	Forward	AAGCCTCGTGCTGTCGGACC
	Reverse	TGAGGCCCAAGGCCACAGG
TNF-α	Forward	CAAGGGACAAGGCTGCCCCG
	Reverse	GCAGGGGCTCTTGACGGCAG
iNOS	Forward	CAGCTGGGCTGTACAAACCTT
	Reverse	CATTGGAAGTGAAGCGTTTCG
CD86	Forward	TCTCCACGGAAACAGCATCT
	Reverse	CTTACGGAAGCACCCATGAT
IL-4	Forward	GAGATGGATGTGCCAAACGTC
	Reverse	CTCTCTGTGGTGTTCTTCGTTG
IL-10	Forward	TAGAGCTGCGGACTGCCTTC
	Reverse	AGAAATCGATGACAGCGCCTC
Arg1	Forward	CAGAAGAATGGAAGAGTCAG
	Reverse	CAGATATGCAGGGAGTCACC
CD206	Forward	TCAGACGAAATCCCTGCTACTG
	Reverse	AGCCTGACCCCAACTTCTCG
IRAK1	Forward	CCACCCTGGGTTATGTGCC
	Reverse	GAGGATGTGAACGAGGTCAGC
TRAF6	Forward	AAAGCGAGAGATTCTTTCCCTG
	Reverse	ACTGGGGACAATTCACTAGAGC
TLR4	Forward	CAAGGGATAAGAACGCTGAGA
	Reverse	GCAATGTCTCTGGCAGGTGTA
GAPDH	Forward	TCCCAGCTTAGGTTCATCAGGT
	Reverse	TACGGGACGAGGAAACACTCTC

### Immunofluorescence Staining

Immunofluorescence staining was performed according to our previously published Protocols (Sun et al., 2014; Ye et al., 2018). Briefly, brain sections (6 μm) were first incubated with primary antibodies against p65 (1:200, 1:1000, cat. No. D14E12, CST), iba-1 (1:200, RRID: AB_2224402) overnight at 4 °C. The next day, brain sections were incubated with corresponding secondary antibodies Alexa Fluor 488 and/or Alexa Fluor 594 (Jackson ImmunoResearch Incorporation, West Grove, PA, United States). Fluorescence was examined under a ZEISS HB050 inverted microscope system. The fluorescently stained cells were analyzed by Image J software.

### Neurotoxicity of Activated Microglia-Conditioned Medium on Neuronal Cells

Neuronal cells were plated in 96-well plates (2 × 10^4) or 24-well plates (2 × 10^5) until maturation at 7 days. Activated microglia-conditioned media (CM) was prepared as follows: (1) the CM from control microglia cells (Control-CM); (2) CM from control+miR-146a group (CM-Con+miR-146a); (3) CM from Hb (20 μM) stimulated microglia for 12 h group (CM-Hb); and (4) CM from Hb (20 μM)+miR-146a group (CM-Hb+miR-146a). Then, the media were transferred to primary neuronal cells. Primary neuronal cell viability and LDH release was assessed after a 12 h incubation by CCK8 assay (Dojindo Laboratories, Japan) and LDH assay (Thermo Scientific, United States) according to the manufacture instructions after 12 h incubation, respectively, according to the manufacturer’s instructions. Changes in the morphology of primary neuronal cells in various groups were observed using a phase-contrast microscope. ROS production was examined by the addition of 10 mM 2,7-dichlorodihydrofluorescein diacetate (DCFH-DA) (Millipore, Sigma) and was observed with green fluorescence under the same exposure times.

### Statistical Analysis

GraphPad Prism (RRID: SCR_002798) for Windows version 7.03 was used to perform statistical analysis. Two-tailed, unpaired Student’s *t*-tests were used to compare two experimental groups. One-way ANOVA followed by *post hoc* Tukey’s tests were performed to determine the difference between each experimental group when comparing three or more groups. Two-way ANOVA was performed to determine the interaction effect of treatments and time courses. *P* < 0.05, *p* < 0.01, and *p* < 0.001 are considered statistically significant, showed as ^∗^ or #, ns: not significant. No assessment of normality of data was carried out. Data of all experiments were included. And therefore, no outlier tests were performed in this study. No statistical methods were applied to predetermine the sample size. Data are expressed as the means ± SD.

## Data Availability Statement

All datasets generated for this study are included in the article/supplementary material.

## Ethics Statement

The animal study was reviewed and approved by the Experimental Animal Ethics Committee of Nanjing Drum Tower Hospital, approved number: 2018020003.

## Author Contributions

G-JL, XG, and HW performed the experiments. C-HH, WL, and Q-RZ contributed to the conception and analysis of the experiments. G-JL, Y-YG, TT, YZ, and X-XC were contributors to writing and editing the manuscript. All authors have read and agreed to the published version of the manuscript.

## Conflict of Interest

The authors declare that the research was conducted in the absence of any commercial or financial relationships that could be construed as a potential conflict of interest.

## Abbreviations

Arg1: arginase 1
CD86: cluster of differentiation 86
CD206: cluster of differentiation 206
EBI: early brain injury
GAPDH: glyceraldehyde-3-phosphate dehydrogenase
Hb: hemoglobin
IF: immunofluorescence
IL-1 β: interleukin 1 beta
IL-4: interleukin 4
IL-10: interleukin 10
iNOS: induced nitric oxide synthases
IRAK1: interleukin-1 receptor-associated kinase 1
LPS: lipopolysaccharide
ROS: reactive oxygen species
RRIDs: research resource identifiers
SAH: subarachnoid hemorrhage
TLR4: Toll-like receptor 4
TNF- α: tumor necrosis factor alpha
TRAF6: TNF receptor associated factor 6.

## References

[B1] Aguzzi A. Barres B. A. Bennett M. L. (2013). Microglia: scapegoat, saboteur, or something else? *Science* 339 156–161. 10.1126/science.1227901 23307732 PMC4431634

[B2] Alam M. M. O’Neill L. A. (2011). MicroRNAs and the resolution phase of inflammation in macrophages. *Eur. J. Immunol.* 41 2482–2485. 10.1002/eji.201141740 21952801

[B3] Aronica E. Fluiter K. Iyer A. Zurolo E. Vreijling J. van Vliet E. A. (2010). Expression pattern of miR-146a, an inflammation-associated microRNA, in experimental and human temporal lobe epilepsy. *Eur. J. Neurosci.* 31 1100–1107. 10.1111/j.1460-9568.2010.07122.x 20214679

[B4] Bi Y. Liu G. Yang R. (2009). MicroRNAs: novel regulators during the immune response. *J. Cell. Physiol.* 218 467–472. 10.1002/jcp.21639 19034913

[B5] Cowan C. Muraleedharan C. K. O’Donnell J. J. 3rd Singh P. K. Lum H. Kumar A. (2014). MicroRNA-146 inhibits thrombin-induced NF-κB activation and subsequent inflammatory responses in human retinal endothelial cells. *Invest. Ophthalmol. Vis. Sci.* 55 4944–4951. 10.1167/iovs.13-13631 24985472

[B6] Doyle S. L. O’Neill L. A. (2006). Toll-like receptors: from the discovery of NFkappaB to new insights into transcriptional regulations in innate immunity. *Biochem. Pharmacol.* 72 1102–1113. 10.1016/j.bcp.2006.07.010 16930560

[B7] East L. (2002). The mannose receptor family. *Biochim. Biophys. Acta* 1572 364–386. 12223280 10.1016/s0304-4165(02)00319-7

[B8] Ferreira T. A. Blackman A. V. Oyrer J. Jayabal S. Chung A. J. Watt A. J. (2014). Neuronal morphometry directly from bitmap images. *Nat. Methods* 11 982–984. 10.1038/nmeth.3125 25264773 PMC5271921

[B9] Fujii M. Yan J. Rolland W. B. Soejima Y. Caner B. Zhang J. H. (2013). Early brain injury, an evolving frontier in subarachnoid hemorrhage research. *Transl. Stroke Res.* 4 432–446. 10.1007/s12975-013-0257-2 23894255 PMC3719879

[B10] Gram M. Sveinsdottir S. Ruscher K. Hansson S. R. Cinthio M. Akerstrom B. (2013). Hemoglobin induces inflammation after preterm intraventricular hemorrhage by methemoglobin formation. *J. Neuroinflamm.* 10:100. 10.1186/1742-2094-10-100 23915174 PMC3750409

[B11] Hanafy K. A. (2013). The role of microglia and the TLR4 pathway in neuronal apoptosis and vasospasm after subarachnoid hemorrhage. *J. Neuroinflamm.* 10:83. 10.1186/1742-2094-10-83 23849248 PMC3750560

[B12] Hanisch U. K. (2013). Functional diversity of microglia – how heterogeneous are they to begin with? *Front. Cell. Neurosci.* 7:65. 10.3389/fncel.2013.00065 23717262 PMC3653062

[B13] Hu X. Leak R. K. Shi Y. Suenaga J. Gao Y. Zheng P. (2015). Microglial and macrophage polarization-new prospects for brain repair. *Nat. Rev. Neurol.* 11 56–64. 10.1038/nrneurol.2014.207 25385337 PMC4395497

[B14] Iyer A. Zurolo E. Prabowo A. Fluiter K. Spliet W. G. van Rijen P. C. (2012). MicroRNA-146a: a key regulator of astrocyte-mediated inflammatory response. *PLoS One* 7:e44789. 10.1371/journal.pone.0044789 23028621 PMC3441440

[B15] Jia L. Wang L. Chopp M. Zhang Y. Szalad A. Zhang Z. G. (2016). MicroRNA 146a locally mediates distal axonal growth of dorsal root ganglia neurons under high glucose and sildenafil conditions. *Neuroscience* 329 43–53. 10.1016/j.neuroscience.2016.05.005 27167084 PMC4905785

[B16] Kwon M. S. Woo S. K. Kurland D. B. Yoon S. H. Palmer A. F. Banerjee U. (2015). Methemoglobin is an endogenous toll-like receptor 4 ligand-relevance to subarachnoid hemorrhage. *Int. J. Mol. Sci.* 16 5028–5046. 10.3390/ijms16035028 25751721 PMC4394463

[B17] Lan X. Han X. Li Q. Yang Q. W. Wang J. (2017). Modulators of microglial activation and polarization after intracerebral haemorrhage. *Nat. Rev. Neurol.* 13 420–433. 10.1038/nrneurol.2017.69 28524175 PMC5575938

[B18] Li S. H. Chen L. Pang X. M. Su S. Y. Zhou X. Chen C. Y. (2017). Decreased miR-146a expression in acute ischemic stroke directly targets the Fbxl10 mRNA and is involved in modulating apoptosis. *Neurochem. Int.* 107 156–167. 10.1016/j.neuint.2017.01.011 28202285

[B19] Lian H. Roy E. Zheng H. (2016). Protocol for primary microglial culture preparation. *Bio Protoc.* 6:e1989. 10.21769/BioProtoc.1989 29104890 PMC5669279

[B20] Lin S. Yin Q. Zhong Q. Lv F. L. Zhou Y. Li J. Q. (2012). Heme activates TLR4-mediated inflammatory injury via MyD88/TRIF signaling pathway in intracerebral hemorrhage. *J. Neuroinflamm.* 9:46. 10.1186/1742-2094-9-46 22394415 PMC3344687

[B21] Lu Y. Zhang X. S. Zhang Z. H. Zhou X. M. Gao Y. Y. Liu G. J. (2018). Peroxiredoxin 2 activates microglia by interacting with Toll-like receptor 4 after subarachnoid hemorrhage. *J. Neuroinflamm.* 15:87. 10.1186/s12974-018-1118-4 29554978 PMC5859544

[B22] Ma X. Becker Buscaglia L. E. Barker J. R. Li Y. (2011). MicroRNAs in NF-kappaB signaling. *J. Mol. Cell. Biol.* 3 159–166. 10.1093/jmcb/mjr007 21502305 PMC3104013

[B23] Miller B. A. Turan N. Chau M. Pradilla G. (2014). Inflammation, vasospasm, and brain injury after subarachnoid hemorrhage. *Biomed. Res. Int.* 2014:384342. 10.1155/2014/384342 25105123 PMC4106062

[B24] Quinn S. R. O’Neill L. A. (2011). A trio of microRNAs that control Toll-like receptor signalling. *Int. Immunol.* 23 421–425. 10.1093/intimm/dxr034 21652514

[B25] Rahimifard M. Maqbool F. Moeini-Nodeh S. Niaz K. Abdollahi M. Braidy N. (2017). Targeting the TLR4 signaling pathway by polyphenols: a novel therapeutic strategy for neuroinflammation. *Ageing Res. Rev.* 36 11–19. 10.1016/j.arr.2017.02.004 28235660

[B26] Rey C. Nadjar A. Buaud B. Vaysse C. Aubert A. Pallet V. (2016). Resolvin D1 and E1 promote resolution of inflammation in microglial cells in vitro. *Brain Behav. Immun.* 55 249–259. 10.1016/j.bbi.2015.12.013 26718448

[B27] Saba R. Sorensen D. L. Booth S. A. (2014). MicroRNA-146a: a dominant, negative regulator of the innate immune response. *Front. Immunol.* 5:578. 10.3389/fimmu.2014.00578 25484882 PMC4240164

[B28] Sehba F. A. (2014). Rat endovascular perforation model. *Transl. Stroke Res.* 5 660–668. 10.1007/s12975-014-0368-4 25213427 PMC4214882

[B29] Sheedy F. J. Palsson-McDermott E. Hennessy E. J. Martin C. O’Leary J. J. Ruan Q. (2010). Negative regulation of TLR4 via targeting of the proinflammatory tumor suppressor PDCD4 by the microRNA miR-21. *Nat. Immunol.* 11 141–147. 10.1038/ni.1828 19946272

[B30] Shi H. Hua X. Kong D. Stein D. Hua F. (2019). Role of Toll-like receptor mediated signaling in traumatic brain injury. *Neuropharmacology* 145 259–267. 10.1016/j.neuropharm.2018.07.022 30075158

[B31] Sison S. L. Patitucci T. N. Seminary E. R. Villalon E. Lorson C. L. Ebert A. D. (2017). Astrocyte-produced miR-146a as a mediator of motor neuron loss in spinal muscular atrophy. *Hum. Mol. Genet.* 26 3409–3420. 10.1093/hmg/ddx230 28637335

[B32] Sun Q. Dai Y. Zhang X. Hu Y. C. Zhang D. Li W. (2013). Expression and cell distribution of myeloid differentiation primary response protein 88 in the cerebral cortex following experimental subarachnoid hemorrhage in rats: a pilot study. *Brain Res.* 1520 134–144. 10.1016/j.brainres.2013.05.010 23684713

[B33] Sun Q. Wu W. Hu Y. C. Li H. Zhang D. Li S. (2014). Early release of high-mobility group box 1 (HMGB1) from neurons in experimental subarachnoid hemorrhage in vivo and in vitro. *J. Neuroinflamm.* 11:106. 10.1186/1742-2094-11-106 24924349 PMC4107626

[B34] Taganov K. D. Boldin M. P. Chang K. J. Baltimore D. (2006). NF-kappaB-dependent induction of microRNA miR-146, an inhibitor targeted to signaling proteins of innate immune responses. *Proc. Natl. Acad. Sci. U.S.A.* 103 12481–12486. 10.1073/pnas.0605298103 16885212 PMC1567904

[B35] Tao T. Liu G. J. Shi X. Zhou Y. Lu Y. Gao Y. Y. (2019). DHEA attenuates microglial activation via induction of JMJD3 in experimental subarachnoid haemorrhage. *J. Neuroinflamm.* 16 243. 10.1186/s12974-019-1641-y 31779639 PMC6883548

[B36] Vasa-Nicotera M. Chen H. Tucci P. Yang A. L. Saintigny G. Menghini R. (2011). miR-146a is modulated in human endothelial cell with aging. *Atherosclerosis* 217 326–330. 10.1016/j.atherosclerosis.2011.03.034 21511256

[B37] Wang L. Chopp M. Szalad A. Zhang Y. Wang X. Zhang R. L. (2014). The role of miR-146a in dorsal root ganglia neurons of experimental diabetic peripheral neuropathy. *Neuroscience* 259 155–163. 10.1016/j.neuroscience.2013.11.057 24316060 PMC3901076

[B38] Wang L. L. Huang Y. Wang G. Chen S. D. (2012). The potential role of microRNA-146 in Alzheimer’s disease: biomarker or therapeutic target? *Med. Hypotheses* 78 398–401. 10.1016/j.mehy.2011.11.019 22209051

[B39] Wang Y. Ge P. Zhu Y. (2013). TLR2 and TLR4 in the brain injury caused by cerebral ischemia and reperfusion. *Mediators Inflamm.* 2013:124614. 10.1155/2013/124614 23864765 PMC3706022

[B40] Xie Y. F. Shu R. Jiang S. Y. Liu D. L. Ni J. Zhang X. L. (2013). MicroRNA-146 inhibits pro-inflammatory cytokine secretion through IL-1 receptor-associated kinase 1 in human gingival fibroblasts. *J. Inflamm. (Lond.)* 10:20. 10.1186/1476-9255-10-20 23680172 PMC3660163

[B41] Ye Z. N. Wu L. Y. Liu J. P. Chen Q. Zhang X. S. Lu Y. (2018). Inhibition of leukotriene B4 synthesis protects against early brain injury possibly via reducing the neutrophil-generated inflammatory response and oxidative stress after subarachnoid hemorrhage in rats. *Behav. Brain Res.* 339 19–27. 10.1016/j.bbr.2017.11.011 29133197

[B42] Zhang J. Zhang Z. G. Lu M. Wang X. Shang X. Elias S. B. (2017). MiR-146a promotes remyelination in a cuprizone model of demyelinating injury. *Neuroscience* 348 252–263. 10.1016/j.neuroscience.2017.02.029 28237816

[B43] Zhang X. S. Li W. Wu Q. Wu L. Y. Ye Z. N. Liu J. P. (2016). Resveratrol attenuates acute inflammatory injury in experimental subarachnoid hemorrhage in rats via inhibition of TLR4 pathway. *Int. J. Mol. Sci.* 17:E1331. 10.3390/ijms17081331 27529233 PMC5000728

